# Exploring fine-scale human and livestock movement in western Kenya

**DOI:** 10.1016/j.onehlt.2019.100081

**Published:** 2019-02-10

**Authors:** Jessica R. Floyd, Nick W. Ruktanonchai, Nicola Wardrop, Andrew J. Tatem, Joseph Ogola, Eric M. Fèvre

**Affiliations:** a WorldPop, Geography and Environmental Science, University of Southampton, Southampton, UK; b Flowminder Foundation, Stockholm, Sweden; c International Livestock Research Institute, Old Naivasha Road, PO BOX 30709, 00100 Nairobi, Kenya; d Institute of Infection and Global Health, University of Liverpool, Leahurst Campus, Neston, UK

## Abstract

Human and livestock mobility are key factors in the transmission of several high-burden zoonoses such as rift valley fever and trypanosomiasis, yet our knowledge of this mobility is relatively poor due to difficulty in quantifying population-level movement patterns. Significant variation in the movement patterns of individual hosts means it is necessary to capture their fine-scale mobility in order to gain useful knowledge that can be extrapolated to a population level. Here we explore how the movements of people and their ruminants, and their exposure to various types of land cover, correlate with ruminant ownership and other demographic factors which could affect individual exposure to zoonoses. The study was conducted in Busia County, western Kenya, where the population are mostly subsistence farmers operating a mixed crop/livestock farming system. We used GPS trackers to collect movement data from 26 people and their ruminants for 1 week per individual in July/August 2016, and the study was repeated at the end of the same year to compare movement patterns between the short rainy and dry seasons respectively. We found that during the dry season, people and their ruminants travelled further on trips outside of the household, and that people spent less time on swampland compared to the short rainy season. Our findings also showed that ruminant owners spent longer and travelled further on trips outside the household than non-ruminant owners, and that people and ruminants from poorer households travelled further than people from relatively wealthier households. These results indicate that some individual-level mobility may be predicted by season and by household characteristics such as ruminant ownership and household wealth, which could have practical uses for assessing individual risk of exposure to some zoonoses and for future modelling studies of zoonosis transmission in similar rural areas.

## 1 Background

Zoonoses cause substantial morbidity and mortality in human and animal populations across the world, threatening less economically developed countries due to the lack of resources needed to efficiently detect and respond to disease events. Because a significant proportion of people are dependent on livestock for their livelihoods in the rural areas where many of the world's poorest communities reside, these diseases are a major factor in perpetuating poverty [[1], [2], [3]]. Zoonotic transmission is ultimately dependent on contact between hosts, whether direct human-to-animal contact or indirect contact through, for example, environmental contamination or vector-borne transmission [4,5]. Zoonoses that are spread directly from host to host include zoonotic influenza and rabies, others like taeniasis/cysticercosis and brucellosis are primarily spread through contact with infected animal products; and vector-borne zoonoses such as trypanosomiasis and Rift Valley Fever (RVF) can be spread though the bite of infected insects. All these types of contact are driven by the movements of human and animal hosts, since contact (and therefore, pathogen movement) is intrinsically dependent on host movement [6].

We know that environmental factors are important in the emergence and maintenance of many zoonotic diseases [4,7,8]. However, suitable environmental conditions are important but not sufficient for zoonotic disease spread, as dynamic host populations must also interact through movement and behaviour to facilitate transmission. Host movements across landscapes can lead to the emergence of pathogens in new hosts and environments and contribute to maintenance of endemicity in old ones [9,10]. Thus, many studies have examined host movement between environments in relation to zoonotic disease spread. For example, vector-borne disease transmission is highly heterogeneous amongst both individual hosts and the landscapes that they move in [[10], [11], [12]] and outbreaks of vector-borne diseases such as RVF are known to be sensitive to both host movement and landscape characteristics, with factors such as host densities and movement patterns contributing to disease maintenance [10,13].

Understanding the movements of people and their livestock, how they interact with their environments and how these correlate with population characteristics could provide crucial information to aid the control of zoonoses, yet few studies have measured these movements simultaneously. Human mobility is important to zoonotic disease risk because it informs where people may spend time with livestock or wild animal populations, and animal mobility is important because it informs where animals may be a risk of exposure from other animals or the environment, but transmission ultimately occurs when hosts meet, whether directly or indirectly. By measuring mobility simultaneously, it is possible to map when, where, and during what kinds of mobility animal-human interactions occur for both populations. For example, knowledge of how people and their livestock move together could help quantify the amount of contact between them, which is a risk factor for transmission of zoonoses such as *Cryptosporidium spp.* [14] and zoonotic *Escherichia coli* [15]. Moreover, the land utilisation of hosts is also related to exposure to some zoonoses: for example, the time spent on different types of land has been shown to affect individual risk of infection of Crimean-Congo haemorrhagic fever (CCHF) [12], and the home ranges of individual hosts have been used to predict threshold host density for the control of foot and mouth disease in Australia [16]. Knowledge of host movement patterns therefore could aid the design of more targeted interventions and behavioural modifications that take into account high risk activities for zoonosis transmission.

Here we present an exploratory study with the aim of measuring the movement patterns of humans and ruminants in a region of western Kenya where several important zoonoses including trypanosomiasis, cysticercosis and Q fever have been previously shown to be endemic [[17], [18], [19]]. We collected GPS data (satellite-informed locations taken at regular intervals) from humans and their ruminants. From these, we calculated measures including the home ranges of individuals, the time they spent on different types of land, and the time spent, distance travelled and frequency of trips outside of the household. By exploring how mobility varies between populations with different demographic characteristics, the study shows how GPS data can provide useful information on their movement patterns. Further studies using similar methods could verify the results shown here and thus help inform how future zoonotic disease interventions are targeted.

## 2 Methods

### 2.1 Study area and sampling procedure

**Fig. 1:**
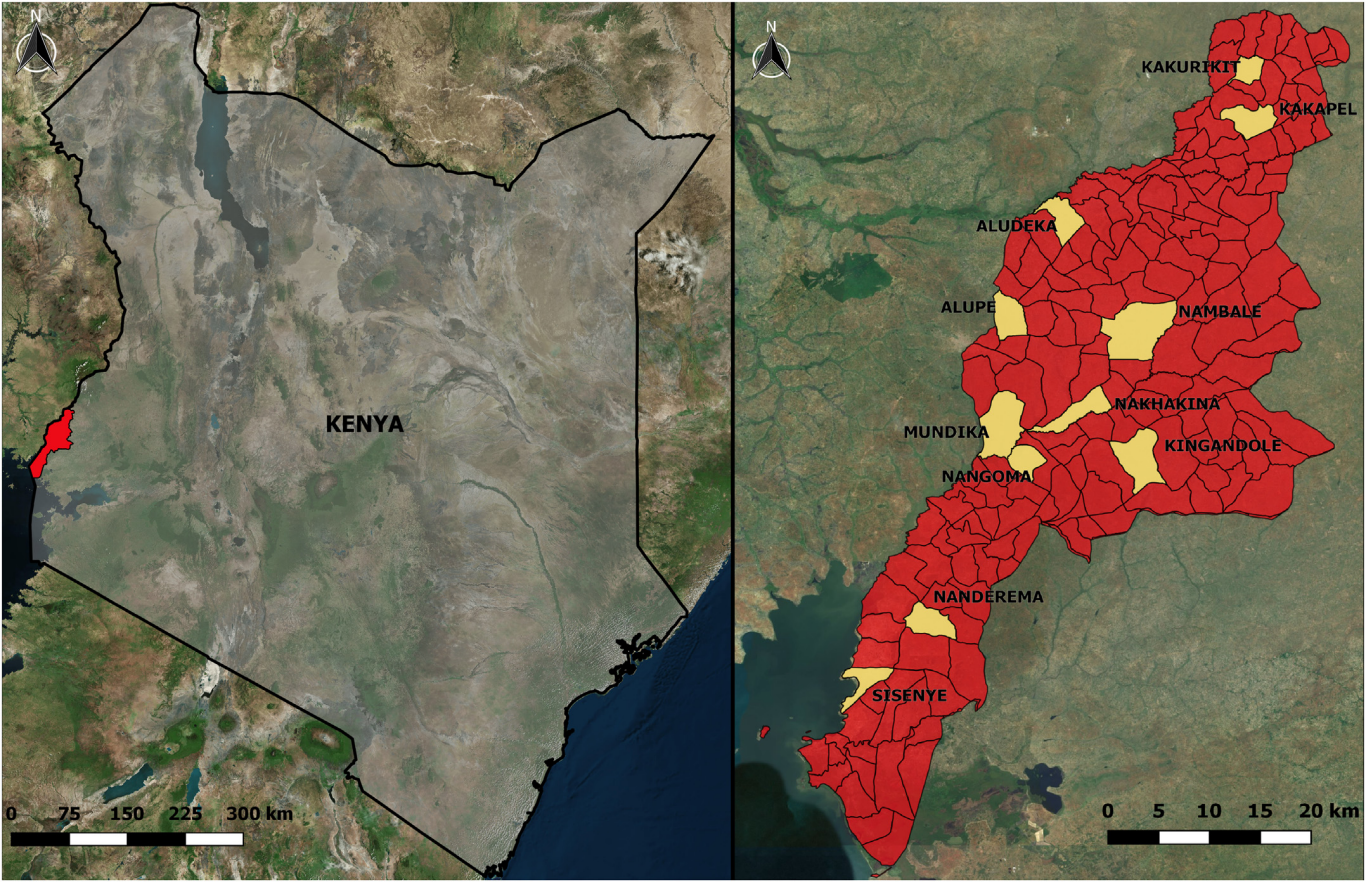
Map showing study location. Left: Kenya with Busia County highlighted in red. Right: Busia County with the sublocations selected for the study in yellow.

The study was conducted in Busia County (Fig. 1) in the Lake Victoria basin region of western Kenya. The human population is just over 800,000 people [20] mainly composed of subsistence farmers operating mixed crop-livestock farming systems [21]. According to the latest DHS survey, 89% of people in Busia live in a rural area (as defined by the national census) and 83% of rural households own livestock [22]. Of these, 60% own cattle [22] usually kept on a mixture of tethered and free grazing systems on common grazing lands [23].

We used clustered random sampling to select 55 households within Busia County. Of the 181 smallest administrative units within the county (known as sublocations), 11 were selected at random to obtain data from a broad geographical range. Within these we visited five households for participation in the study to optimise the use of the limited number of GPS trackers. In the absence of adequate household density data, we used random generation of geographical coordinates within each sublocation followed by identification of the household closest to the coordinates within 200 m to choose the households, using protocol established by other studies in this area [21]. This method will necessarily result in bias toward selection of rural households, which we deemed acceptable given the high proportion of rural households in Busia County. We conducted the survey at all study households and the GPS tracking at 26 randomly chosen of the 55 originally selected households (all of whom agreed to participate), in 6 of the 11 sublocations. The adult who spent the most time tending to the ruminants was asked to wear the tracker, or, if there were no ruminants, the household head. See the supplementary information for details.

### 2.2 Data collection

The survey (see Supplementary information) was administered to the consenting adults in each household individually. The questions addressed the following: (a) basic demographic and household information, (b) an individual's movements and (c) their activities involving livestock. The survey allowed estimation of the relative wealth of households by scoring the answers to 10 standardized questions using the 2011 Poverty Probability Index for Kenya [24].

For the GPS data collection, participants were given a GPS tracker (i-gotU GT-600, accuracy of 25 m [25]) fitted to a lanyard for 1 week, and asked to wear it during the daytime. Simultaneously, if the household kept ruminants then one of these (preferably a cow or bull) was chosen through random number generation to wear an identical waterproofed GPS tracker on an adjustable collar. If the household kept no ruminants, only the participant was given a tracker to allow for comparison of movement patterns between people with and without ruminants. At the end of the week, the researchers returned to the household to collect the trackers and download the data.The household survey and GPS tracking were conducted in short rainy season (July/August 2016) and the GPS tracking was repeated during the dry season (November/December 2016) with the same participants where possible, to capture potential differences in movement patterns during different seasons. These seasons are named in accordance with climate classifications for this region of Kenya [26].

### 2.3 Data analysis

Data were mapped in QGIS (v2.18 [27]) for inspection, then cleaned and analysed in R software (v3.4.2 [28]) using the *trip* [29], *lme4* [30] and *glmmTMB* [31] packages. A linear interpolation algorithm was used to clean the data and calculate one point per minute over the collection period. From these data, we calculated five movement measurements for each subject: the time spent, maximum distance travelled and frequency of trips outside of the household, the home range of the subject and the time spent on different types of landcover. A trip was defined as the time when the location of a subject was recorded 100 m or farther from their household for >15 min. Home ranges were calculated using the minimum convex polygon method. Land cover data for the region were obtained from Wardrop et al. [32] and used to calculate the time spent by people and ruminants on each of 5 types of land cover in the classification: artificial/bare land (defined in the classification as non-vegetated land), crops/grassland, woodland/shrubs, rice paddies and swampland.

We first conducted univariable analyses using linear regression for the four movement measures (time spent, maximum distance travelled and frequency of trips outside of the household), and beta regression for the proportion of time spent in different types of land cover. The covariates used, given in Table 1, were selected from covariates used in a previous study of a zoonosis in this region [19] and had *a priori* plausibility. We log transformed the data for time spent on trips, maximum distance travelled and home range to ensure an approximately normal distribution and used linear models to examine their relationships with the covariates. We then combined the statistically significant factors (*p* < .05) from the univariable analyses into a multivariable model to quantify their impact in context of each other. We also tested for interactions between season and statistically significant covariates. Because of the hierarchical nature of the data (individuals nested in clusters, with repeat measurements per individual), we used linear mixed models (LMMs) and generalized LMMs (GLMMs) for the multivariable analyses. All mixed models had the individual household nested within the sublocation as a random effect, to account for variation between individuals from different households and within different sublocations. When analysing time spent on different types of land as the measured outcome, we calculated the number of minutes spent on and off each of the land types, and then used a beta family in a GLMM to obtain odds ratios for the different effects (see Supplementary information).

**Table 1:** Individual and household characteristics used in analyses.

Demographic covariates	Number of participants: Survey & GPS	Number of participants: GPS only
Gender		
Male	33 (43.4%)	18 (69.2%)
Female	43 (56.6%)	8 (30.8%)
Age		
18–29	22	7
30–49	26	8
50–69	23	7
70+	5	4
Main occupation		
Farming/agriculture	45	18
Hunting	2	2
Trading	3	1
Other	18	4
Unemployed	8	1
Relative wealth score (PPI Kenya^a^) of participant's household		
<30	16	9
30 to 50	24	9
51 or more	12	8
Ruminant ownership of participant's household		
No ruminants	12	6
Ruminants	40	20

a PPI Kenya = Poverty Probability Index for Kenya 2011, see Supplementary information for details.

## 3 Results

GPS data were successfully collected from 26 humans and 20 ruminants from 26 households in the first season, and from 25 humans and 15 ruminants in the second season (87% of the original tracked individuals), for a total of 86 unique GPS readings. Three of the ruminant GPS units were damaged on retrieval and the data could not be extracted, two suffered battery issues and one was irretrievable. Table 1 provides some characteristics of participating households.

### 3.1 Movements beyond the household

**Fig. 2:**
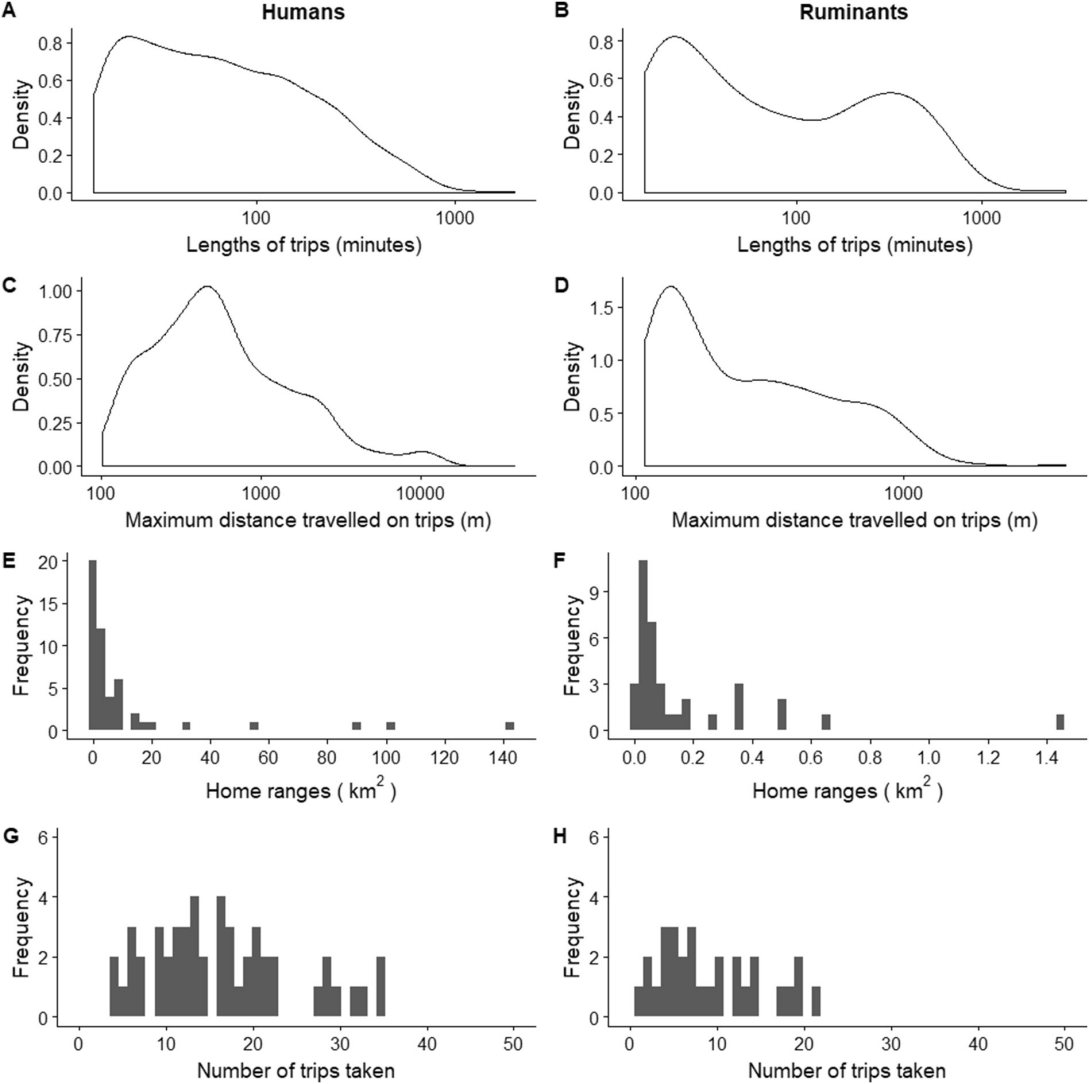
Summary of the human (A,C,E,G) and ruminant (B,D,F,H) GPS data. A,B Lengths of trips outside the household (*n* = 934, *n* = 306). C,D Maximum distance travelled on trips outside the household (*n* = 934, *n* = 306). E,F Home ranges (*n* = 51, *n* = 36). G,H Frequency of trips outside the household (*n* = 51, *n* = 29).

Humans spent a mean of 2.4 h per trip outside of the household while their ruminants spent 4.7 h. Humans travelled significantly further than their ruminants on these trips, with a mean maximum distance of 1060 m travelled compared to 362 m for ruminants. They also had larger home ranges, with a mean area of 7.5 km^2^ compared to 0.1 km^2^ for ruminants. Finally, humans took more frequent trips away from the household, with a mean of 18 trips per week compared to 9 for ruminants (Fig. 2).

Exploratory analyses using univariable linear regression with random effects showed that some demographic covariates had statistically significant (*p* < .05) effects on the movement response variables (Table 2). Ruminant owners spent longer and travelled further on trips outside of the household than non-owners. We also observed a link between household wealth and distance travelled on trips, with people travelling 0.9 times farther per 10-point increase in their household wealth. For ruminants, a similar pattern was found: the wealth of the household was associated with both the time spent and frequency of trips taken by ruminants on trips outside of the household, with animals from poorer households spending more time outside of the household and taking more frequent trips. Differences in trip frequency were not associated with any of the covariates tested for humans.

**Table 2:** Univariable linear regression for movement response variables. The time spent, maximum distance and home ranges were log transformed before modelling, thus these estimates are factor increases and decreases, while estimates for trip frequency are absolute. For ruminants, only the relevant covariates were tested.

Response variable	Explanatory variable	Estimate	*p*-value
Time spent on trips outside of the household (humans)	Ruminant ownership: yes [Ref = no]	1.47 [1.24, 1.76]	<.001⁎⁎⁎
	Number of ruminants	1.12 [1.04, 1.20]	.002⁎⁎
	Gender: male [Ref = female]	1.07 [0.91, 1.26]	.396
	Occupation: non-farmer [Ref = farmer]	1.02 [0.86, 1.20]	.836
	Season: dry [Ref = short rainy]	1.21 [1.05, 1.39]	.008⁎⁎
	Household wealth	1.00 [1.00, 1.00]	.768
	Age (years)	1.01 [1.00, 1.01]	<.001⁎⁎⁎
Time spent on trips outside of the household (ruminants)	Number of ruminants	1.32 [1.04, 1.67]	.023⁎
	Season: dry [Ref = short rainy]	0.95 [0.72, 1.27]	.735
	Household wealth	0.99 [0.98, 1.00]	.008⁎⁎
Maximum distance travelled outside of the household (humans)	Ruminant ownership: yes [Ref = no]	1.50 [1.25, 1.80]	<.001⁎⁎⁎
	Number of ruminants	1.10 [1.02, 1.18]	.015⁎
	Gender: male [Ref = female]	1.15 [0.97, 1.37]	.107
	Occupation: non-farmer [Ref = farmer]	1.21 [1.02, 1.44]	.034 ⁎
	Season: dry [Ref = short rainy]	1.32 [1.16, 1.51]	<.001⁎⁎⁎
	Household wealth	0.99 [0.99, 1.00]	<.001⁎⁎⁎
	Age (years)	1.00 [1.00, 1.01]	.158
Maximum distance travelled outside of the household (ruminants)	Number of ruminants	1.09 [0.94, 1.26]	.246
	Season: dry [Ref = short rainy]	0.99 [0.85, 1.15]	.848
	Household wealth	0.99 [0.99, 1.00]	.040⁎
Home range (humans)	Ruminant ownership: yes [Ref = no]	3.31 [0.81, 14.43]	.111
	Number of ruminants	1.20 [0.64, 2.24]	.572
	Gender: male [Ref = female]	1.60 [0.41, 6.28]	.501
	Occupation: non-farmer [Ref = farmer]	1.33 [0.36, 5.54]	.677
	Season: dry [Ref = short rainy]	3.28 [1.58, 7.04]	.004⁎⁎
	Household wealth	0.99 [0.95, 1.02]	.428
	Age (years)	1.03 [1.00, 1.07]	.049⁎
Home range (ruminants)	Number of ruminants	2.09 [1.06, 4.12]	.049⁎
	Season: dry [Ref = short rainy]	0.77 [0.43, 1.35]	.368
	Household wealth	1.01 [0.98, 1.04]	.623
Trip frequency (total number of trips taken, humans)	Ruminant ownership: yes [Ref = no]	1.87 [−5.11, 8.71]	.594
	Number of ruminants	1.05 [−1.96, 3.94]	.486
	Gender: male [Ref = female]	4.90 [−2.73, 10.96]	.126
	Occupation: non-farmer [Ref = farmer]	−2.10 [−8.33, 4.14]	.517
	Season: dry [Ref = short rainy]	−1.11 [−4.79, 2.67]	.557
	Household wealth	0.00 [−0.16, 0.16]	.990
	Age (years)	−0.05 [−0.22, 0.11]	.513
Trip frequency (total number of trips taken, ruminants)	Number of ruminants	1.82 [−1.19, 5.88]	.285
	Season: dry [Ref = short rainy]	1.82 [−2.22, 5.60]	.360
	Household wealth	0.14 [0.00, 0.26]	.038⁎

Figures in square brackets are 95% confidence intervals.

⁎⁎⁎ *p* < .001.

⁎⁎ *p* < .01.

⁎ *p* < .05.

**Fig. 3:**
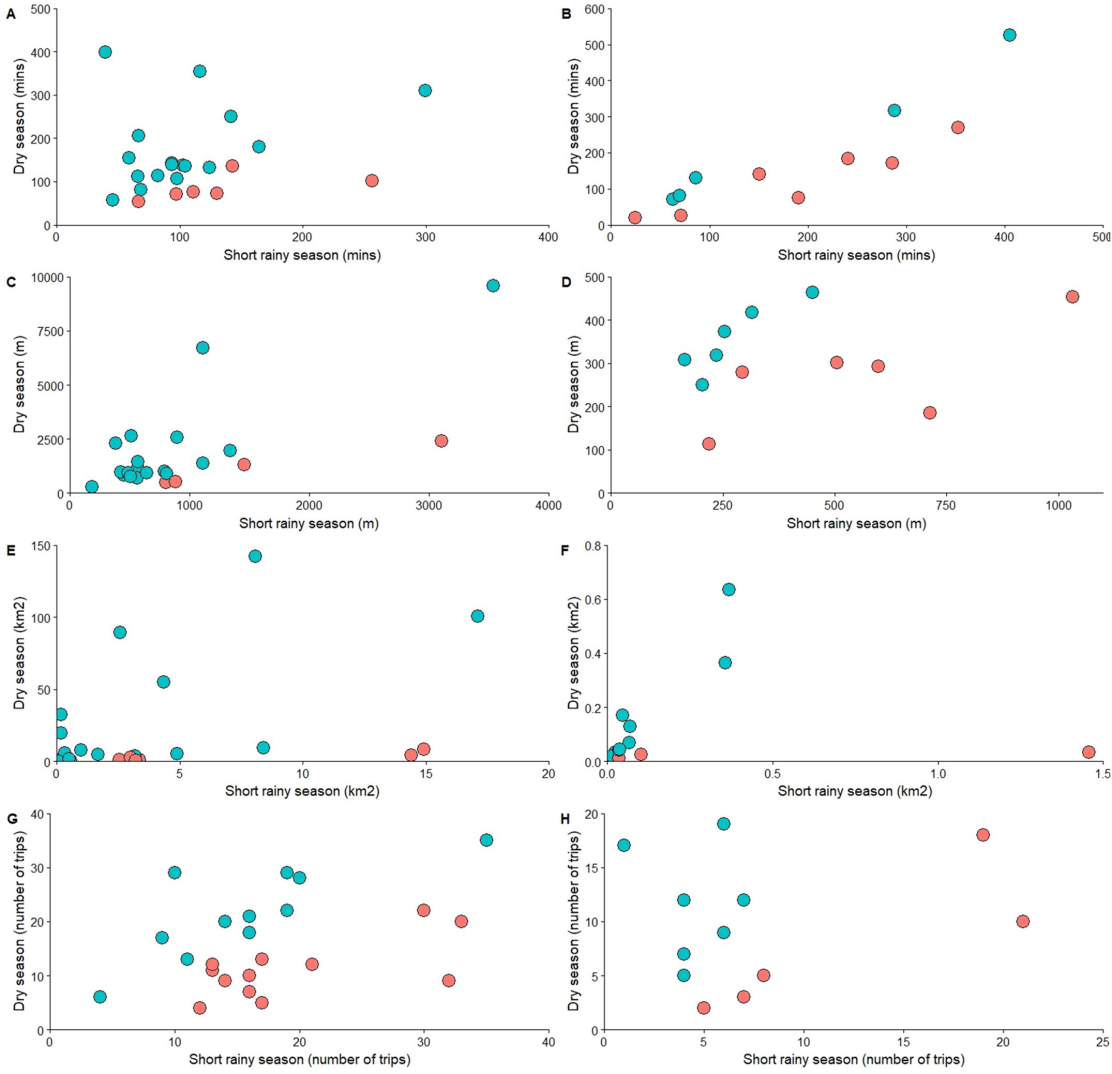
Comparison of movement measures between seasons for humans (A,C,E,G) and ruminants (B,D,F,H). Points are blue where the dry season value is larger than in the short rainy season, and red otherwise. A,B Lengths of trips outside of the household. C,D Maximum distance travelled on trips outside of the household. E,F Home ranges. G,H Frequency of trips outside of the household.

We found significant differences between the two seasons in the distances participants travelled on trips and their overall home ranges (Fig. 3). Humans spent 1.21 times longer on trips outside of the household (*p* = .008), travelled 1.32 times further (*p* < .001) and had home ranges that were 3.28 times larger (*p* = .004) in the dry season compared to the short rainy season. No link was found between season and trip frequency. The movement measures did not vary significantly by sublocation in either season, and ruminant movement measures showed no significant differences between the seasons.

Using only the significant covariates from the univariable analyses, we then carried out a multivariable linear regression (see Supplementary information). In these analyses, only season remained a significant covariate, confirming the finding that in the dry season people took longer trips, travelled further on them and had larger home ranges compared to the short rainy season.

We tested for interactions between season and the significant covariates from the univariable analyses, and found that season variable interacted significantly with both ruminant ownership and number of ruminants owned for the time spent and distance travelled outside of the household. Our most relevant finding was that in the short rainy season, ruminant owners spent almost twice as long and travelled 1.67 times further on trips outside of the household (*p* = .002 and .049, respectively) compared to non-owners. Full results are presented in the Supplementary information.

### 3.2 Movements and landcover

In univariable beta regression analyses we found that some of the demographic covariates were associated with time spent on different types of land, including ruminant ownership: ruminant owners spent more time on wood/shrubland compared to non-ruminant owners (OR 2.79). Moreover, the odds of a participant spending time on wood/shrubland were 1.38 times higher for each extra ruminant owned. The full univariable results are presented in the Supplementary information. The time spent by humans and their ruminants on two types of land cover varied significantly with season. During the dry season, people spent less time on swampland compared to the short rainy season, and their ruminants spent more time on artificial/bare land. The time spent on other types of landcover did not vary significantly by season (Table 3). We tested for the interaction of season with ruminant ownership for time spent on different types of land but found few interactions that were significant and relevant to the focus of this paper.

**Table 3:** Comparison of time spent by humans and ruminants on different land types between wet and dry seasons. Univariable beta regression models with sublocation as a random effect.

Land type	Host	Odds ratio (dry season compared to short rainy season)	*p*-value
Artificial/bare	Human	1.12 [0.92, 1.36]	.267
	Ruminant	1.27 [1.03, 1.58]	.028⁎
Crops/grassland	Human	1.01 [0.87, 1.17]	.929
	Ruminant	1.16 [0.90, 1.51]	.256⁎⁎
Rice paddies	Human	1.10 [0.95, 1.26]	.204⁎⁎⁎
	Ruminant	1.00 [0.91, 1.09]	.981
Swamp	Human	0.83 [0.69, 0.99]	.034⁎
	Ruminant	1.25 [0.96, 1.63	.093
Woodland/shrubs	Human	0.96 [0.81, 1.14]	.642
	Ruminant	1.31 [0.88, 1.95]	.188

Figures in square brackets are 95% confidence intervals.

⁎⁎⁎ *p* < .001.

⁎⁎ *p* < .01.

⁎ *p* < .05

## 4 Discussion

Host movements are critical to the propagation of some of the highest burden zoonoses. The movement patterns that contribute to zoonosis risk are relatively unknown, and there is little understanding of how movements vary between different seasons and population groups for both humans and livestock in the context of zoonotic disease. Although this was an exploratory study limited by a small sample size and biased toward rural households, the seasonal and demographic differences in the movement patterns of people and their ruminants observed indicate that these patterns are worthy of further study.

In the dry season, we found that people took longer trips, travelled further on them and had larger home ranges compared to the short rainy season, yet these measures for their ruminants remained similar across the two seasons. This suggests the extra distance travelled by people during the dry season was not done with their ruminants, which could be because people had to travel further in the dry season to access resources needed for the household, such as water or forage for their livestock. We also found that ruminant owners in both seasons travelled further and for longer than non-owners, supporting this conclusion. This key difference in movement between seasons has been identified in previous studies: a study done in Zambia using similar methods also found that people living in rural areas travelled further during the dry season [33]. However, few disease transmission models incorporate this information, likely due to a lack of understanding of how best to account for these differences in movement.

The link between movement and household wealth indicates that people and ruminants from relatively poorer households travelled further than those from wealthier households in Busia County. This could support the relevance of resource access - if people from wealthier households have the option of accessing resources nearer to their household, they may not make as many long-distance trips as people from poorer households. We know that people from poorer households often travel further to access health facilities [34]; our findings suggest that this may extend to other types of resources, such as water and forage, although further work is needed to verify our findings. A previous study in Busia County has shown that household wealth is linked to ease of resource access and risk of infection, with poorer households having more difficulty accessing resources and being at higher risk of certain zoonoses (unpublished results). Since individual movements are a key factor in zoonotic transmission, it could be that the wider-ranging movement patterns of people and ruminants from poorer households are putting them at additional risk of infection. Further research to identify the types of places people and their herds travel to could pinpoint ‘hotspots’ of disease transmission.

We show that humans spent more time in swampy areas during the short rainy season than the dry. People may be spending more time on swampland in the short rainy season to take advantage of water sources or grazing areas for their ruminants, or potentially as a water source for the household. Swampland is prone to flooding, and studies of mosquito habitats have shown that it tends to have a higher abundance of larval breeding habitats for zoonotic vectors such as *Aedes aegypti* and *Aedes albopictus* as well as malaria vectors (*Anopheles spp.*) than other types of land [35,36]. Therefore, spending more time in swampland in the short rainy season may be exposing both humans and their ruminants to mosquito-borne diseases, though further analysis with a larger sample size is clearly needed to verify this conclusion across these populations.

The use of GPS devices to track people and animals in urban and rural settings has been previously documented [37,38] and shown to be a reliable tool for tracking movements, with good portability, weight and battery life. This exploratory study confirms the feasibility of simultaneously tracking human and livestock movements, as this had not previously been tested. Although the sample size was small, the data collected from participants yielded some interesting results over the two seasons studied. In future work, a larger sample size in this area could provide more detailed conclusions and reveal patterns that are generalizable to similar rural populations, particularly those with livestock. To overcome the bias in household selection, high-resolution population density data like those produced by WorldPop [39] could be used to weight the selection of various points on the map. Nevertheless, the data obtained by tracking individuals across the two seasons were especially valuable, and thus a longitudinal cohort design where individuals are tracked continuously over the year could be an ideal method to gain data on long-term and long-distance movements, thus facilitating estimation of zoonotic disease risk on wider spatial and temporal scales.

## Declarations of interest

We declare no conflicts of interest.

## Appendix A Supplementary data

Supplementary material Image 1
