# Supplementary material for: Exploring fine-scale human and livestock movement in western Kenya
Source: One Health. 2019 Feb 10;7:100081. doi: 10.1016/j.onehlt.2019.100081 (PMC6416412; doi:10.1016/j.onehlt.2019.100081)
Supplement: Supplementary file 1 — Supplementary material [file mmc1.docx]

**Supplementary information 1: Data collection**

- 1. Household selection

A surplus of ten coordinates were generated in each sublocation, so that if a household could not be identified within 200 metres, the next generated coordinates were used. QGIS software tools were used for the random selection of sublocations and coordinates. We chose to sample 55 households from a broad geographical range based on the manpower and time available for fieldwork, while optimising the use of the limited number of GPS trackers, due to a lack of appropriate data on which to base a sample size calculation.

Households were selected if they were the main residence of at least one consenting adult present at the time of the visit. All households visited were asked to participate in the survey, and half of these were also asked to participate in the GPS tracking. Inclusion criteria for participation were consenting adults aged 18 or over who were present at the time of the visit. If the household declined to participate in the study, the next household closest to the coordinates was visited. All adults within a household were selected for participation in the survey, but only one was selected for participation in the GPS tracking: this was the adult who spent the most time looking after the ruminants, determined by a combination of survey responses and a verbal discussion of household roles. If the household had no ruminants, the head of the household (determined by verbal communication) was selected instead.

1.2 Survey

The survey was administered to the consenting adults in each household, and consisted of up to 516 questions, a subset of which were asked depending on the individual being questioned (household-specific questions were only asked once per household) and the number and types of livestock kept by the household. The survey was conducted through a trained interpreter in Kiswahili, Dholuo, Luhya or English depending on the language choice of the individual being interviewed. Data were recorded on a handheld tablet loaded with the survey written with OpenDataKit software. The data were later uploaded to a secure cloud-based server once an internet connection was established.

1.3 GPS data

*GPS devices*

The iGotU GT-600 GPS tracking device (Mobile Action Technology Inc., Taiwan) was used for the GPS tracking element of the study; it has a long battery life (up to 10 days of tracking on battery saver mode), high portability and small size and weight. Previous studies using an earlier model of this device have found it to be highly accurate for human and livestock movement tracking and socially acceptable in certain settings [1,2]. Humans were able to wear the device on a lanyard or in a pocket, while for animals the device was attached to a homemade collar made of nylon webbing and a simple plastic buckle.

The tracker recorded a GPS location once every 30 seconds while the participant was moving at a speed of 10km/h or more, and once a minute otherwise. Battery saver mode was enabled so that when the wearer was not moving at all the tracker would turn off, for example overnight. The data were downloaded from the tracker using the ‘@trip’ software provided by Mobile Action with the trackers. The data were stored on a password-protected laptop and external hard drive.

**Supplementary information 2: Data analysis**

2.1 Integrity of the GPS data

For humans and ruminants, respective means of 162.90 (range 64.60 to 222.80) and 139.70 (range 55.02 to 192.20) hours of data were collected during the short rainy season, with the difference for ruminants attributed to the need for battery recalibration early in the season. The GPS units were set to capture one point a minute while the wearer was active. On average the units collected 26 points per hour, with a range of 7 to 36 for human and 51 points per hour with a range of 25 to 59 for ruminants. The difference between human and ruminant points per minute was mainly due to the fact that humans could take their unit off while sleeping, meaning no points were recorded during this time.

Approximately 167.40 (range 45.53 to 210.00) hours of data for humans and 165.10 (range 135.30 to 191.60) hours for ruminants were collected during the dry season. The GPS units were set to capture one point a minute while the wearer was active. More points were collected by humans during the short rainy season (26 points per hour, range 7 to 36) than in the dry season (22 points per hour, range 8 to 35), (paired t test, *p*-value = 0.011), which may indicate more frequent movement by humans in the short rainy season or may simply be due to lower compliance by humans wearing the trackers the second time they were visited. There was no significant difference (paired t test, *p* = 0.23) in the number of points recorded per hour for ruminants (51 points per hour, range 25 to 59 in the short rainy season versus 47 points per hour, range 33 to 52 in the dry season).

2.2 Data cleaning

A linear interpolation algorithm was used to generate one point per minute for minutes where no points were recorded during the time the subject kept the GPS unit. This facilitated calculation of the amount of time spent in a given area or on a trip outside the household, as well as comparisons of these measures between households. Previous studies have found this method of linear interpolation to be satisfactory with regards to accuracy and precision at estimating GPS locations. Anomalous points identified by their unlikely speeds and trajectories were excluded before the implementation of this algorithm.

The relative wealth scores of the households were calculated using the 2011 Poverty Probability Index (PPI) for Kenya: this is a poverty measurement tool that is designed to estimate the likelihoods that a household is living below various poverty lines by scoring the answers to ten Kenya-specific questions [3], which were included in the survey. The raw wealth scores from this tool were used in the analysis without converting them into poverty likelihoods, since we used this tool for comparison of wealth between the households, rather than to make statements about whether a household is below a particular poverty line.

2.3 Univariable and multivariable analyses

The *stats* [4] package in R version 3.4.2 was used to perform univariable linear regression of the covariates against the five movement measures. The *adehabitatHR* [5] package was used to calculate the home range of each participant using the minimum convex polygon method. The *lme4* [6] and *glmmTMB* [7] packages were used to construct linear mixed models of relationships between movement measurements and various fixed effects, which included ruminant ownership, relative household wealth, gender and occupation. The household sublocation and individuals nested within sublocations were given as random effects.

The statistically significant (p < 0.05) variables identified in the univariable analyses of the movement response variables were used to construct a multivariable linear regression model (Supplementary Table 1). These variables were also tested for interaction with season and the results are presented in Supplementary Table 3.

Supplementary Table 3 shows the univariable beta regression for the amount of time spent by humans and ruminants on different types of land, using the beta family in a generalized linear mixed model. Supplementary Table 4 shows the tests for interaction of the ruminant ownership variable with the season variable. Since there were few significant variables in the univariable analyses, only ruminant ownership was tested for interaction with season as we believed it had the highest *a priori* plausibility.

| **Response variable** | **Explanatory variable** | **Estimate** | **p-value** |
| --- | --- | --- | --- |
| Time spent on trips outside of the household (humans) | Ruminant ownership: yes [Ref = no] | 0.92 [0.52, 1.87] | 0.788 |
|  | Number of ruminants | 1.17 [0.91, 1.46] | 0.198 |
|  | Season: dry [Ref = short rainy] | 1.23 [1.07, 1.41] | 0.003 ** |
|  | Age | 1.01 [1.00, 1.01] | 0.171 |
| Time spent on trips outside of the household (ruminants) | Number of ruminants  Household wealth | 0.95 [0.62, 1.46]  1.00 [0.98, 1.01] | 0.804  0.648 |
| Maximum distance travelled outside of the household (humans) | Ruminant ownership: yes [Ref = no] | 1.35 [0.56, 2.98] | 0.494 |
|  | Number of ruminants | 1.09 [0.78, 1.53] | 0.644 |
|  | Occupation: non-farmer [Ref = farmer] | 1.37 [0.88, 2.19] | 0.201 |
|  | Season: dry [Ref = short rainy] | 1.38 [1.21, 1.57] | < 0.001 *** |
|  | Household wealth | 1.00 [0.99, 1.01] | 0.628 |
| Home range (humans) | Season: dry [Ref = short rainy] | 3.20 [1.55, 6.83] | 0.004 ** |
|  | Age | 1.03 [1.00, 1.07] | 0.056 |

**Supplementary Table 1 | Multivariable linear regression for movement response variables.** The time spent, maximum distance and home ranges were log transformed before modelling, thus these estimates are factor increases and decreases. Figures in square brackets are 95% confidence intervals. *** p < 0.001, ** p < 0.01, * p < 0.05.

| **Response variable** | **Interactions tested** | **Estimate** | **p-value** |
| --- | --- | --- | --- |
| Time spent on trips outside of the household (humans) | Ruminant ownership: yes [Ref = no]  Season: dry [Ref = short rainy]  Ruminant ownership: yes *Season: dry | 1.88 [1.32, 2.69]  2.12 [1.53, 2.94]  0.52 [0.36, 0.74] | 0.002 **  < 0.001 ***  < 0.001 *** |
|  | Number of ruminants  Season: dry [Ref = short rainy]  Number of ruminants*Season: dry | 1.29 [1.12, 1.51]  1.77 [1.37, 2.29]  0.79 [0.69, 0.91] | 0.002 **  < 0.001 ***  0.001 ** |
|  | Age  Season: dry [Ref = short rainy]  Age*Season: dry [Ref = short rainy] | 1.01 [1.00, 1.01]  0.96 [0.63, 1.44]  1.01 [1.00, 1.01] | 0.235  0.836  0.197 |
| Time spent on trips outside of the household (ruminants) | Number of ruminants  Season: dry [Ref = short rainy]  Number of ruminants*Season: dry | 0.95 [0.66, 1.38]  0.78 [0.49, 1.24]  1.09 [0.72, 1.65] | 0.789  0.292  0.689 |
|  | Household wealth  Season: dry [Ref = short rainy]  Household wealth*Season: dry | 1.00 [0.98, 1.01]  0.82 [0.41, 1.67]  1.00 [0.98, 1.02] | 0.756  0.578  0.940 |
| Maximum distance travelled outside of the household (humans) | Ruminant ownership: yes [Ref = no]  Season: dry [Ref = short rainy]  Ruminant ownership: yes *Season: dry | 1.67 [1.02, 2.73]  1.95 [1.42, 2.66]  0.66 [0.47, 0.93] | 0.049 *  < 0.001 ***  0.018 * |
|  | Number of ruminants  Season: dry [Ref = short rainy]  Number of ruminants*Season: dry | 1.22 [0.99, 1.50]  1.70 [1.33, 2.17]  0.88 [0.77, 1.00] | 0.078  < 0.001 ***  0.054 |
|  | Occupation: non-farmer [Ref = farmer]  Season: dry [Ref = short rainy]  Occupation: non-farmer *Season: dry | 1.31 [0.82, 2.13]  1.45 [1.25, 1.69]  0.83 [0.62, 1.13] | 0.271  < 0.001 ***  0.243 |
|  | Household wealth  Season: dry [Ref = short rainy]  Household wealth*Season: dry | 0.99 [0.98, 1.01]  1.22 [0.87, 1.72]  1.00 [1.00, 1.01] | 0.366  0.251  0.430 |
| Maximum distance travelled outside of the household (ruminants) | Household wealth  Season: dry [Ref = short rainy]  Household wealth*Season: dry | 1.01 [1.00, 1.01]  1.30 [0.86, 1.99]  0.99 [0.98, 1.00] | 0.183  0.216  0.098 |
| Home range (humans) | Age  Season: dry [Ref = short rainy]  Age*Season: dry | 1.03 [0.99, 1.07]  2.46 [0.32, 20.63]  1.01 [0.96, 1.05] | 0.133  0.406  0.794 |
| Home range (ruminants) | Number of ruminants  Season: dry [Ref = short rainy]  Number of ruminants*Season: dry | 2.29 [1.10, 4.75]  1.47 [0.22, 9.50]  0.72 [0.28, 1.84] | 0.040  0.697  0.504 |

**Supplementary Table 2 | Univariable linear regression for movement response variables including interaction terms for significant covariates.** The time spent, maximum distance and home ranges were log transformed before modelling, thus these estimates are factor increases and decreases. Figures in square brackets are 95% confidence intervals. *** p < 0.001, ** p < 0.01, * p < 0.05.

| **Land type** | **Explanatory variable** | **Odds ratio** | **p-value** |
| --- | --- | --- | --- |
| **Artificial or bare land (humans)** | Ruminant ownership: yes [Ref = no] | 1.63 [0.36, 7.37] | 0.527 |
|  | Number of ruminants | 0.93 [0.63, 1.38] | 0.727 |
|  | Gender: male [Ref = female] | 1.08 [0.32, 3.68] | 0.900 |
|  | Occupation: non-farmer [Ref = farmer] | 1.21 [0.36, 4.05] | 0.758 |
|  | Season: dry [Ref = short rainy] | 1.12 [0.92, 1.36] | 0.267 |
|  | Household wealth | 0.97 [0.95, 1.00] | 0.093 |
|  | Age | 1.02 [0.99, 1.05] | 0.193 |
| **Artificial or bare land (ruminants)** | Number of ruminants | Invalid model | NA |
|  | Season: dry [Ref = short rainy] | 1.27 [1.03, 1.58] | 0.028 * |
|  | Household wealth | 0.96 [0.92, 1.00] | 0.041 * |
| **Crops or grassland (humans)** | Ruminant ownership: yes [Ref = no] | 0.74 [0.46, 1.17] | 0.196 |
|  | Number of ruminants | 0.98 [0.85, 1.13] | 0.773 |
|  | Gender: male [Ref = female] | 0.87 [0.57, 1.35] | 0.545 |
|  | Occupation: non-farmer [Ref = farmer] | 1.45 [0.94, 2.22] | 0.091 |
|  | Season: dry [Ref = short rainy] | 1.01 [0.87, 1.17] | 0.929 |
|  | Household wealth | 1.01 [1.00, 1.02] | 0.152 |
|  | Age | 1.00 [0.98, 1.01] | 0.459 |
| **Crops or grassland (ruminants)** | Number of ruminants | 1.00 [0.75, 1.34] | 0.991 |
|  | Season: dry [Ref = short rainy] | 1.16 [0.90, 1.51] | 0.256 |
|  | Household wealth | 1.01 [0.99, 1.02] | 0.215 |
| **Rice paddies (humans)** | Ruminant ownership: yes [Ref = no] | 1.07 [0.87, 1.32] | 0.493 |
|  | Number of ruminants | 1.05 [1.00, 1.11] | 0.073 |
|  | Gender: male [Ref = female] | 0.96 [0.81, 1.13] | 0.633 |
|  | Occupation: non-farmer [Ref = farmer] | 0.94 [0.80, 1.12] | 0.492 |
|  | Season: dry [Ref = short rainy] | 1.10 [0.95, 1.26] | 0.204 |
|  | Household wealth | Invalid model | NA |
|  | Age | 1.00 [0.99, 1.00] | 0.541 |
| **Rice paddies (ruminants)** | Number of ruminants | 1.08 [1.03, 1.13] | 0.001 ** |
|  | Season: dry [Ref = short rainy] | 1.00 [0.91, 1.09] | 0.981 |
|  | Household wealth | 1.00 [1.00, 1.00] | 0.518 |
| **Swampland (humans)** | Ruminant ownership: yes [Ref = no] | 1.19 [0.62, 2.27] | 0.609 |
|  | Number of ruminants | 1.19 [0.89, 1.57] | 0.240 |
|  | Gender: male [Ref = female] | 1.57 [0.86, 2.85] | 0.141 |
|  | Occupation: non-farmer [Ref = farmer] | 0.45 [0.18, 1.10] | 0.079 |
|  | Season: dry [Ref = short rainy] | 0.83 [0.69, 0.99] | 0.034 * |
|  | Household wealth | 1.01 [0.99, 1.04] | 0.385 |
|  | Age | 0.99 [0.96, 1.01] | 0.273 |
| **Swampland (ruminants)** | Number of ruminants | 1.68 [0.83, 3.42] | 0.149 |
|  | Season: dry [Ref = short rainy] | 1.25 [0.96, 1.63] | 0.093 |
|  | Household wealth | 1.01 [0.98, 1.04] | 0.688 |
| **Woodland or shrubs (humans)** | Ruminant ownership: yes [Ref = no] | 2.79 [1.11, 6.98] | 0.029 * |
|  | Number of ruminants | 1.38 [1.06, 1.80] | 0.017 * |
|  | Gender: male [Ref = female] | 1.17 [0.49, 2.82] | 0.718 |
|  | Occupation: non-farmer [Ref = farmer] | 0.94 [0.38, 2.34] | 0.901 |
|  | Season: dry [Ref = short rainy] | 0.96 [0.81, 1.14] | 0.642 |
|  | Household wealth | 1.01 [0.99, 1.04] | 0.315 |
|  | Age | 1.00 [0.98, 1.03] | 0.746 |
| **Woodland or shrubs (ruminants)** | Number of ruminants | 1.32 [0.89, 1.98] | 0.173 |
|  | Season: dry [Ref = short rainy] | 1.31 [0.88, 1.95] | 0.188 |
|  | Household wealth | 1.02 [1.00, 1.04] | 0.067 |

**Supplementary Table 3 | Univariable beta regression for time spent by humans and ruminants on different types of land.** Figures in square brackets are 95% confidence intervals. *** p < 0.001, ** p < 0.01, * p < 0.05.

| **Land type** | **Explanatory variable** | **Odds ratio** | **p-value** |
| --- | --- | --- | --- |
| **Artificial or bare land** | Ruminant ownership: yes [Ref = no]  Season: dry [Ref = short rainy]  Ruminant ownership: yes *Season: dry | 2.89 [0.63, 13.33]  3.97 [1.50, 10.53]  0.30 [0.11, 0.84] | 0.173  0.006 **  0.023 * |
| **Crops or grassland** | Ruminant ownership: yes [Ref = no]  Season: dry [Ref = short rainy]  Ruminant ownership: yes *Season: dry | 0.88 [0.60, 1.30]  1.08 [0.78, 1.50]  0.88 [0.61, 1.27] | 0.529  0.630  0.501 |
| **Rice paddies** | Ruminant ownership: yes [Ref = no]  Season: dry [Ref = short rainy]  Ruminant ownership: yes *Season: dry | 1.29 [0.47, 3.55]  1.72 [0.51, 5.81]  0.76 [0.19, 2.94] | 0.619  0.381  0.686 |
| **Swampland** | Ruminant ownership: yes [Ref = no]  Season: dry [Ref = short rainy]  Ruminant ownership: yes *Season: dry | 2.94 [0.77, 11.30]  2.19 [0.98, 4.87]  0.36 [0.15, 0.84] | 0.116  0.056  0.018 * |
| **Woodland or shrubs** | Ruminant ownership: yes [Ref = no]  Season: dry [Ref = short rainy]  Ruminant ownership: yes *Season: dry | Invalid model | NA |

**Supplementary Table 4 | Univariable beta regression for time spent by humans on different types of land including interaction between ruminant ownership and season.** Figures in square brackets are 95% confidence intervals. *** p < 0.001, ** p < 0.01, * p < 0.05.

**Supplementary information 3: Household Survey**

**Individual questions**

- Sublocation
- Household ID
- Collect the GPS coordinates of this household.
- Language of administration
- Name of respondent
- Age of respondent
- Gender of respondent
- Tribal origin
- Principal religion
- Marital status
- Level of education reached
- Years lived in current village
- Major occupation

**Individual movement questions**

Do you regularly visit any of these places?

- School
- Place of work (livestock-related)
- Place of work (other)
- Health facility
- Place of worship
- Market - animal
- Market - other
- Shop
- Another household
- Less regular, e.g. annual visits

For each place:

- Does the place have a name?
- How often do you typically go there?
- How do you usually travel there?
- How much does it cost?
- How long does it take you to get there (minutes)?
- How long do you typically spend there (hours)?
- Do any children go with you?
- How many?

**Household questions**

- How many members does this household have?
- Gender of household member
- Age of household member
- What is the highest school grade that the female head/spouse has completed?
- What is the main occupation of the male head/spouse?
- How many habitable rooms does this household occupy in its main dwelling?
- What is the floor of the main dwelling predominantly made of?
- What is your source of water for cooking?
- What is your source of water for drinking?
- What is the main source of lighting fuel for the household?
- Is there a latrine in the household?
- What type(s)?
- Is there evidence of scrounging by animals around the latrine?
- Transport: does your household own any of the following?
- Does your household own any irons (charcoal or electric?)
- How many mosquito nets does your household own?
- How many towels does your household own?
- How many frying pans does your household own?
- Do you grow crops?
- Why do you grow crops?
- How do you get to your crops?
- How much does it cost?
- Approximately how long does it take you to get to your crops in minutes?
- Where does the majority of your household access medical facilities?
- How do you normally travel to the medical facility?
- How long does it normally take to get there (minutes)?

**Household livestock questions**

- Does this homestead keep any animals?
- Where do you access veterinary services?
- Have you used the veterinary services in the last 12 months?
- Does this homestead keep cattle?
- Cattle questions
- How many males?
- How many females?
- Why do you keep cattle?
- Do you ever buy cattle or have you received cattle as a gift from outside the household?
- How long ago did you last buy/receive new cattle?
- Where do you usually buy/receive new cattle from?
- Do your cows/bulls engage in communal breeding?
- Are cattle herded with goats or sheep?
- How do you graze/feed your cattle?
- What is the water source for your cattle?
- Do you use medicine to prevent or treat disease in your cattle?
- Where do you get medicine for your cattle from?
- Have any cattle in the home been given any vaccinations?
- Do you know which vaccines have been given?
- Do you always pasteurize your milk before consumption?

**Cattle questions**

- Are you involved in feeding the cattle?
- Are you involved in milking the cattle?
- Are you involved in taking cattle to water?
- Are you involved in birthing the cattle?
- Have you ever experienced abortion in your herd?
- When was the last abortion?
- Do you ever handle cattle abortion material?
- What do you do with the aborted material?
- Are you involved in handling cattle manure?
- Do you ever buy cattle from a market?
- Do you ever take the cattle to a market?
- Are you involved in cattle skinning?
- Are you involved in cattle burial?
  - For each of the above:
  - How often do you do this activity?
  - Do you have to travel outside the household to do this activity?
  - What type of place do you go to for this activity?
  - If yes, how do you travel there?
  - How much does it cost?
  - How long does it take you to travel there (minutes)?
  - How long do you spend there (hours)?
  - How many children go with you?
  - What is the name of the place?

**Pig questions**

- Does this homestead keep pigs?
- How many piglets?
- How many adult males?
- How many adult females?
- Why do you keep pigs?
- Do you buy pigs from a market?
- What is the name of the market?
- How do you normally travel to the market?
- How much does it cost?
- How long does it normally take to get there (minutes)?
- How do you house the pigs?
- How do you feed the pigs?
- Are pigs fed waste?
- If pigs are fed waste, is it cooked prior to feeding it to the pig?
- Are pigs housed during any season?
- What is the flooring in the pig housing?
- Do you use medicine to prevent or treat disease in your pigs?
- Where do you purchase medicine for your pigs?
- Have the pigs on the homestead been vaccinated against anything?
- Does the participant know which vaccine was given to the pigs?
- Name/purpose of vaccine
- Are there any significant problems with your pigs?
- Are you involved in feeding the pigs?
- Are you involved in taking pigs to water?
- Are you involved in birthing the pigs?
- Are you involved in handling pig manure?
- Are you involved in taking pigs to market?
- Are you involved in pig slaughter?
- Are your pigs inspected at slaughter?
- Who inspects the meat for cysts?
- If cysts are found, what do you do with the meat?
- Are you involved in pig burial?
- For each of the activities above:
  - How often do you do this activity?
  - Do you have to travel outside the household to do this activity?
  - What type of place do you go to for this activity?
  - If yes, how do you travel there?
  - How much does it cost?
  - How long does it take you to travel there (minutes)?
  - How long do you spend there (hours)?
  - How many children go with you?
  - What is the name of the place?

**Sheep/goat questions**

- Does this homestead keep sheep/goats?
- How many sheep/goats?
- Do you buy sheep/goats from a market?
- What is the name of the market?
- How do you normally travel to the market?
- How much does it cost?
- How long does it normally take to get there (minutes)?
- How do you house the sheep/goats?
- How do you graze/feed your sheep/goats?
- Are you involved in feeding the sheep/goats?
- Are you involved in milking the sheep/goats?
- Are you involved in taking sheep/goats to water?
- Are you involved in birthing the sheep/goats?
- Are you involved in handling sheep/goat abortion material?
- Are you involved in handling sheep/goats manure?
- Are you involved in taking sheep/goats to market?
- Are you involved in sheep/goat slaughter?
- Are you involved in sheep/goat skinning?
- Are you involved in sheep/goat burial?
- For each of the activities above:
  - How often do you do this activity?
  - Do you have to travel outside the household to do this activity?
  - What type of place do you go to for this activity?
  - If yes, how do you travel there?
  - How much does it cost?
  - How long does it take you to travel there (minutes)?
  - How long do you spend there (hours)?
  - How many children go with you?
  - What is the name of the place?

**Final questions**

- Does this homestead keep chickens?
- Do you feed your chickens?
- Do livestock have access to the buildings you sleep in?
- Which livestock have access to the buildings you sleep in?
- In the last 12 months, have you seen wildlife around the home?
- What wildlife have you seen?
- Tracker given?
- Tracker ID

**References**

[1] V. A. Paz-Soldan *et al.*, “Strengths and Weaknesses of Global Positioning System (GPS) Data-Loggers and Semi-structured Interviews for Capturing Fine-scale Human Mobility: Findings from Iquitos, Peru,” *PLoS Negl. Trop. Dis.*, vol. 8, no. 6, p. e2888, Jun. 2014.

[2] M. B. Parsons *et al.*, “Global positioning system data-loggers: a tool to quantify fine-scale movement of domestic animals to evaluate potential for zoonotic transmission to an endangered wildlife population.,” *PLoS One*, vol. 9, no. 11, p. e110984, Jan. 2014.

[3] “Kenya | PPI,” *Poverty Probability Index*, 2011. [Online]. Available: https://www.povertyindex.org/country/kenya. [Accessed: 23-Feb-2018].

[4] R. C. Team, “R: A language and environment for statistical computing.” R Foundation for Statistical Computing, Vienna, Austria, 2017.

[5] C. Calenge, “The package adehabitat for the R software: a tool for the analysis of space and habitat use by animals.,” *Ecol. Modell.*, vol. 197, pp. 516–519, 2006.

[6] S. W. Douglas Bates, Martin Maechler, Ben Bolker, “Fitting Linear Mixed-Effects Models Using lme4.,” *J. Stat. Softw.*, vol. 67, no. 1, pp. 1–48, 2015.

[7] M. E. Brooks *et al.*, “glmmTMB Balances Speed and Flexibility Among Packages for Zero-inflated Generalized Linear Mixed Modeling,” *R J.*, vol. 9, no. 2, pp. 378–400, 2017.
